# Biodegradable Hydrogenated Dimer Acid-Based Plasticizers for PLA with Excellent Plasticization, Thermal Stability and Gas Resistance

**DOI:** 10.3390/molecules29112526

**Published:** 2024-05-27

**Authors:** Nengkun Huang, Fan Wang, Ruihao Zhang, Zhaolin Cao, Wen Sun, Yuting Ma, Jihuai Tan, Xinbao Zhu

**Affiliations:** 1 Jiangsu Co-Innovation Center of Efficient Processing and Utilization of Forest Resources, College of Chemical Engineering, Nanjing Forestry University, Nanjing 210037, China; 2 College of Light Industry and Food Science, Nanjing Forestry University, Nanjing 210037, China

**Keywords:** vegetable oil-based plasticizer, hydrogenated dimer acid, PLA, thermal stability, biodegradability

## Abstract

The use of vegetable oil-dervied plasticizers to enhance the flexibility of polylactic acid (PLA) has received much attention due to their renewability, inexpensiveness and biodegradation. However, the double bonds in vegetable oil-based plasticizers limit their compatibility with PLA, resulting in PLA-derived products with reduced flexibility. Herein, we examined soybean oil-derived hydrogenated dimer acid-based polyethylene glycol methyl ether esters (HDA-2*n*, 2*n* = 2, 4, 6 or 8, referring to the ethoxy units) developed via the direct esterification of saturated hydrogenated dimer acid and polyethylene glycol monomethyl ethers. The resulting HDA-2*n* was first used as a plasticizer for PLA, and the effects of the ethoxy units in HDA-2*n* on the overall performance of the plasticized PLA were systematically investigated. The results showed that, compared with PLA blended with dioctyl terephthalate (DOTP), the PLA plasticized by HDA-8 with the maximum number of ethoxy units (PLA/HDA-8) exhibited better low-temperature resistance (40.1 °C vs. 15.3 °C), thermal stability (246.8 °C vs. 327.6 °C) and gas barrier properties. Additionally, the biodegradation results showed that HDA-8 could be biodegraded by directly burying it in soil. All results suggest that HDA-8 could be used as green alternative to the traditional petroleum-based plasticizer DOTP, which is applied in the PLA industry.

## 1. Introduction

Polylactic acid (PLA), as a biodegradable polymer, has gained extensive attention because of its outstanding mechanical properties, biocompatibility and processability, as well as its optical clarity, and it has been widely used in food packaging [1], textiles [2], medical devices [3] and automobile manufacturing [4]. However, the intrinsic brittleness of PLA limits its further application, and it must be modified to meet industrial requirements. To date, various approaches, including copolymerization modification [5], blending modification [6] and plasticization modification [7] methods, have been explored to overcome the brittleness of PLA. Among them, the utilization of a plasticizer to enhance the flexibility of PLA is a feasible method for industrial applications due to its simplicity, cost and efficiency. Previous studies have demonstrated that polyethylene glycol [8], polypropylene glycol [9], dioctyl phthalate [10] and polybutyl acrylate [11] can efficiently improve the flexibility of PLA; nevertheless, most of these plasticizers come from non-renewable petroleum resources.

Natural vegetable oils are considered ideal raw materials for the preparation of bio-based plasticizers due to their wide availability, non-toxicity and biodegradability [12,13]. A series of ester-branched plasticizers were developed through the ring-opening reaction of epoxidized soybean oil and acid anhydride, and they were used as environmentally friendly plasticizers for PVC to completely replace di-(2-ethylhexyl) phthalate (DOP) [14]. The results indicated that its migration resistance, low cytotoxicity and biodegradability were better than DOP. Quiles-Carrillo et al. [15] synthesized acrylated epoxidized soybean oil (AESO) using epoxidized soybean and acrylic acid as feedstocks, and compared to neat PLA, the toughness and mechanical properties of the PLA blended with the AESO were significantly increased, which could be attributed to the chain extension and/or cross-linking process of the biopolymer chains, which was facilitated by the presence of the acrylate and epoxy groups in their structures. However, most of these vegetable oleic acids belong to linear fatty acids, with longer alkyl carbon chains and fewer polar groups, which are not conducive to the development of high-quality vegetable oil-based plasticizers; in addition, the residual double bonds in the structures of vegetable oil-based plasticizers not only affect their stability at high temperature but also cause poor compatibility and poor weather resistance [16,17].

In contrast, dimer acids contain binary carboxyl groups, which make it possible to introduce more polar groups, and their ring structure and high molecular weight significantly improve the heat resistance and migration resistance of the derived plasticizer. Lee et al. [18] synthesized alkyl dimer acid esters (DA-C_n_) of different chain lengths from waste soybean oil and used them to plasticize ethyl cellulose (EC). The results showed that the migration resistance and plasticizing properties of the methyl dimer acid (DA-C_1_) were better than those of the traditional plasticizer, DOP. Tan et al. [19] synthesized ethylene glycol dimer acid ether ester (DA-2*n*) with different numbers of ethoxy functional groups and used it to plasticize PVC. The results showed that the DA-8 had migration resistance and thermal stability, which were better than those of the commercial plasticizer DOTP. In addition, Tan et al. [20] utilized high-value monomeric fatty acids derived from dimer acid production and prepared a series of bio-based plasticizers for nitrile butadiene rubber (NBR). These studies mainly focused on non-PLA materials, but the structure of PLA is different from these materials, and the interaction between the hydroxyl and carboxyl groups and a dimer acid-derived plasticizer may be relatively weak, resulting in incompatibility between the plasticizer and the PLA. This lack of compatibility may cause the plasticizer not to interact effectively with the PLA molecules and reduce the plasticizing effect in the PLA, and so the above dimer acid-derived plasticizers are not suitable for plasticizing PLA. What is more important is that the above plasticizer also retains its double bond, which further causes problems such as poor compatibility [21].

The hydrogenation of dimer acid to a hydrogenated dimer acid is a mature industrial behavior, which not only retains the original characteristics of the dimer acid but also makes the product lighter in color, with better stability, and it is more suitable for the preparation of a plasticizer. Liu et al. [22] presented a method for creating fully bio-based and super-tough PLA blends using dynamic vulcanization with a biomass-derived diisocyanate (LDI) and hydrogenated dimer acid (HDA). The study demonstrated that the in situ formation of a cross-linked biopolyamide network (HDAPA) within PLA significantly enhanced the impact toughness and melt flowability of the material. In this study, hydrogenated dimer acid-based polyethylene glycol methyl ether ester (HDA-2*n*, 2*n* = 2, 4, 6 or 8, referring to the ethoxy units) containing a flexible ethoxy functional group was prepared by a one-step esterification using non-toxic and degradable hydrogenated dimer acid as a raw material. We studied the structure–activity relationship of the compound, examined, in detail, the effect of its structure on the properties of the plasticized PLA samples and successfully developed a substitute, which could completely replace the toxic petroleum-based plasticizer, DOTP. Compared with the DOTP-plasticized PLA samples, PLA/HDA-2*n* samples have good compatibility, thermal stability, transparency and biodegradability.

## 2. Results

### 2.1. Structural Characterizations

**Figure 1:**
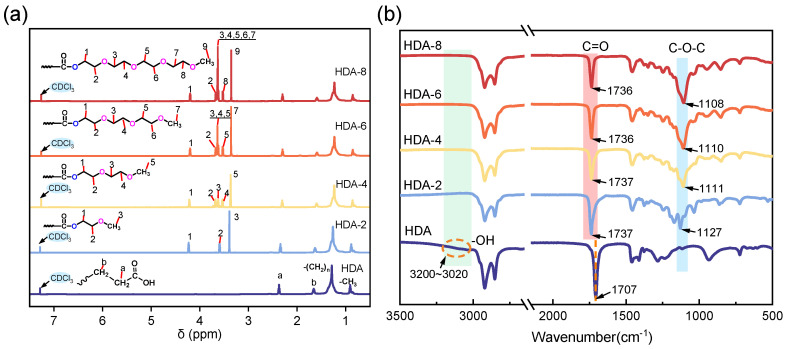
^1^H NMR (**a**) and FTIR (**b**) spectra of HDA and HDA-2*n*.

As depicted in Figure 1a, the ^1^HNMR spectra of hydrogenated dimer acid (HDA) and hydrogenated dimer acid-based polyethylene glycol methyl ether ester (HDA-2*n*, 2*n* = 2, 4, 6, 8) were compared to evaluate the successful esterification between HDA and polyethylene glycol methyl ether. Compared with HDA, in Figure 1a, distinct peaks at 3.5–3.7 ppm emerged, corresponding to the methylene hydrogen associated with the ether bond, observed in the ^1^HNMR spectrum of HDA-2*n*. This observation signifies the successful esterification of hydrogenated dimer acid with alcohol ether, resulting in the formation of the respective alcohol ether ester. Furthermore, new peaks at 4.2 ppm are also shown in Figure 1a, attributed to the hydrogen peak on the methylene connected to the carboxylate, which further suggested the esterification of hydrogenated dimer acid with alcohol ether.

FTIR analysis was further employed to examine the structures of HDA and HDA-2*n*. As shown in Figure 1b, the infrared spectrum of HDA exhibits a broad absorption peak around 3060 cm^−1^, corresponding to the -OH group, while no such peak is evident in the infrared spectrum of hydrogenated dimer acid-based polyethylene glycol methyl ether ester, which implied successful esterification between hydroxyl groups from polyethylene glycol methyl ether ester and the carboxy group of HDA. Furthermore, the infrared spectra of HDA-2*n* revealed a distinctive ester group peak at 1738 cm^−1^ and an identifiable ether bond (C-O-C) peak at 1106 cm^−1^. These observations also confirmed the successful esterification of hydrogenated dimer acid with polyethylene glycol methyl ether ester.

### 2.2. Mechanical Properties and Low-Temperature Resistance of Plasticized PLA Samples

**Figure 2:**
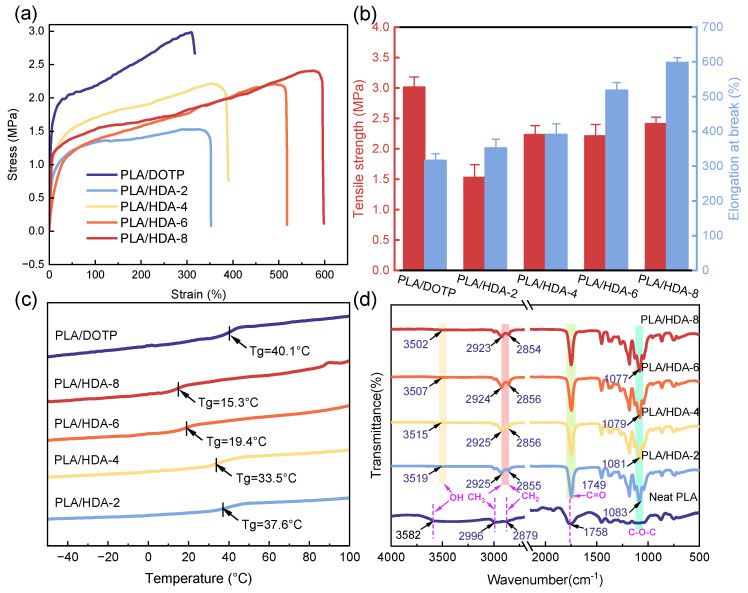
(**a**) Stress–strain curves of plasticized PLA; (**b**) values of tensile strength and elongation at break of plasticized PLA; (**c**) DSC curves of plasticized PLA; (**d**) FTIR of neat and plasticized PLA around 4000~500 cm^−1^.

The tensile characteristics of plasticized PLA can be used as an index to evaluate the plasticizing efficiency. As shown in Figure 2a, the PLA samples plasticized with HDA-2*n* showed typical flexible deformations, demonstrating an effective reduction in PLA’s underlying brittleness. The elongation at the break of plasticized PLA decreased as follows: PLA/HDA-8 > PLA/HDA-6 > PLA/HDA-4 > PLA/HDA-2 > PLA/DOTP. Obviously, PLA-plasticized HDA-2*n* showed a linear increase in elongation at break along with an increase in ethoxy units, outperforming conventional DOTP. It should be noted that the elongation at break of PLA products plasticized using HDA-8 was 281 percent higher than that of DOTP.

The glass transition temperature (*T*_g_) of plasticized PLA products serves as a crucial metric for assessing the plasticizers’ capacity to mitigate molecular chain interactions. *T*_g_ is influenced not only by the plasticizer’s structure but also by its compatibility with PLA [23]. As shown in Figure 2c, the *T*_g_ of HDA-2*n* steadily declines, ranging from 37.6 °C to 15.3 °C. Notably, the *T*_g_ values of PLA/HDA-2 and PLA/HDA-4 were slightly lower than that of PLA/DOTP (*T*_g_ = 40.1 °C), indicating that their plasticizing effect closely resembles that of DOTP. In general, HDA-2*n* exhibits a superior *T*_g_ value compared to the traditional plasticizer, DOTP. This observation suggests that the incorporation of flexible ether bond groups effectively enhances the plasticizer’s properties. The impact of ester groups on the plasticization of HDA-2*n* was further analyzed by FTIR.

As illustrated in Figure 2d, the nonpolar groups (methyl and methylene) as well as the polar carbonyl group in HDA-2*n*-plasticized PLA samples exhibit a shift towards lower wavenumbers compared to neat PLA. In the case of HDA-2*n*-plasticized PLA, the ether bond also underwent a shift from 1127~1108 cm^−1^ (Figure 1b) to 1083 cm^−1^. The shift in the nonpolar methyl and methylene groups could be attributed to their intermolecular interactions (Van der Waals force) with the methyl groups in PLA [24]. Similarly, the movement of the polar carbonyl group and ether bond can be ascribed to their interaction with the hydroxyl group present in the PLA structure, resulting in the formation of hydrogen bonds [25]. Additionally, with an increase in the number of ethoxy functional groups, the wavenumber of the ether bonds steadily decreases. This result suggested a gradual reduction in the attractive forces between PLA chains, which could be attributed to the hydrogen bond interaction between the flexible ethoxy functional groups in HDA-2*n* and the hydroxyl groups in the PLA structure. As a result, the interaction forces between PLA molecules diminished, which aligned with results from the literature [26]. The possible interaction between HDA-2*n* and the PLA matrix is shown in Figure S2. Additionally, the mechanical properties and glass transition temperature of some plasticizers used for plasticizing PLA are summarized in Table 1. As shown in Table 1, the properties of some plasticizers reported in previous research are similar to those of HDA-8 in this work; however, the processes for modification methods in previous works could not easily realize industrial applications.

### 2.3. XRD Spectra Analysis

**Figure 3:**
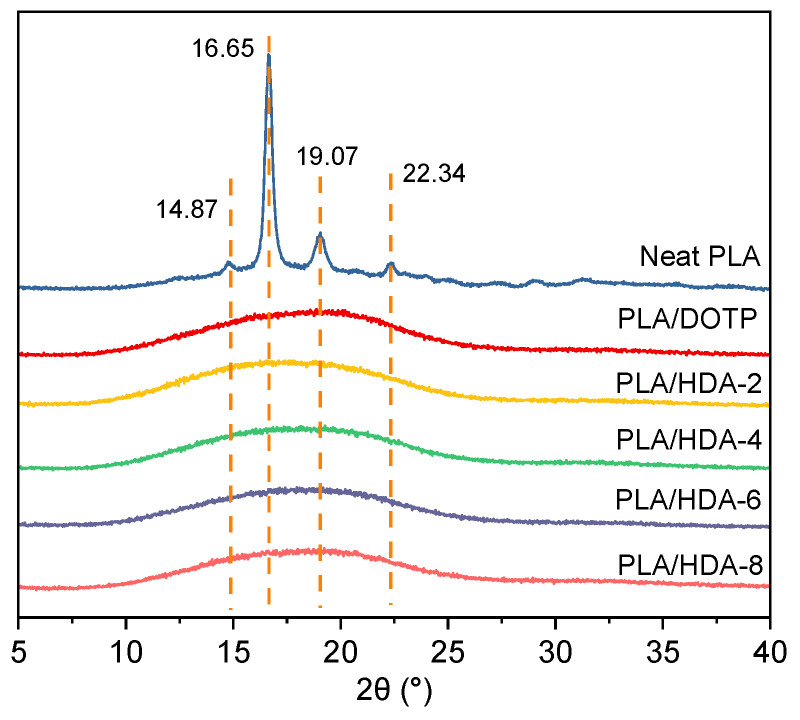
X-ray diffraction spectra of neat PLA and plasticized PLA films.

In order to further study the effect of DOTP and HDA-2*n* on the structure of PLA, neat PLA and plasticized PLA were scanned by X-ray diffraction. As shown in Figure 3, it can be seen that the neat PLA sample prepared by the solvent method has typical α crystal [32] characteristic peaks observed at 14.87°, 16.65°, 19.07° and 22.34°, respectively. The addition of a plasticizer significantly weakens the diffraction peak intensity of the PLA sample. This is because the plasticizer inserted and left in the PLA crystallization area makes the PLA molecules less tightly arranged and disrupts the crystal structure of PLA. The diffraction peak intensity of PLA/HDA-8 is weaker than that of PLA/DOTP, indicating that the ability of HDA-8 in reducing the crystallinity of PLA was better than DOTP, delivering better plasticizing properties for PLA.

### 2.4. Surface Analysis of Plasticized PLA Samples

**Figure 4:**
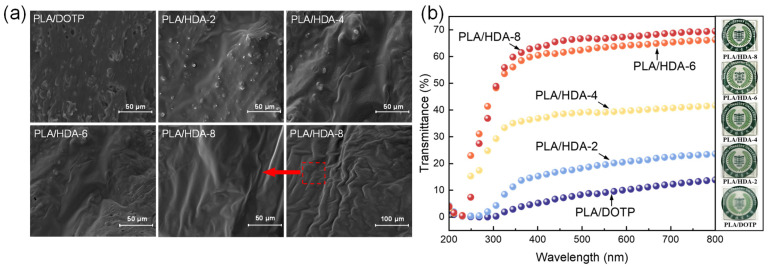
(**a**) SEM images of plasticized PLA samples; (**b**) UV-vis spectra of PLA samples.

The fracture surfaces of plasticized PLA samples were examined using a scanning electron microscope, and the results are presented in Figure 4a. Blending PLA with DOTP, HDA-2 and HDA-4 resulted in uneven surfaces and numerous small particles in the samples, likely attributed to their low compatibility. Conversely, PLA samples plasticized with HDA-6 and HDA-8 exhibited comparatively flat surfaces, with PLA/HDA-8, in particular, demonstrating the smoothest and most uniform surface. This can be attributed to the fact that its structure contains the most polar ethoxy units, which promotes the formation of interaction with PLA molecules and significantly enhances the compatibility in the PLA matrix. As a result, its surface exhibits the greatest uniformity and smoothness, a characteristic linked to its ductile fracture behavior [33]. This observation aligned with the findings from DSC analysis.

Transparency is another important property for the PLA product, which determines the application aspects. In this work, the transparency of plasticized PLA products was tested by UV spectroscopy, and photos of the PLA samples are shown, as shown in Figure 4b. From the photos, the transparency of PLA/HDA-2*n* is obviously better than that of PLA/DOTP, which indicates that the compatibility of HDA-2*n* with a PLA carrier is better than that of the traditional plasticizer, DOTP. With an increase in the number of ethoxy units, the transparency of plasticized PLA samples increased continuously, which was also consistent with a previous report [34].

### 2.5. Thermal Stability and Migration Resistance of Plasticized PLA

**Figure 5:**
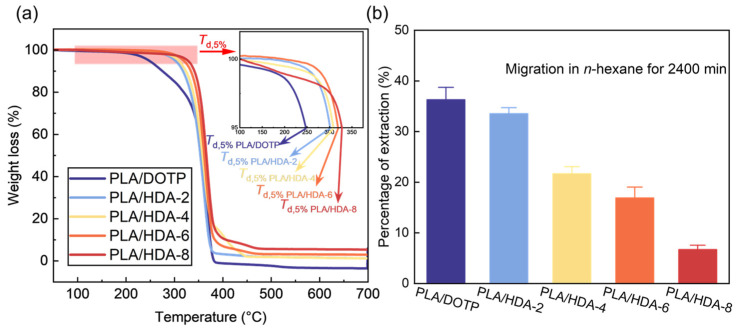
(**a**) Thermal stability of plasticized PLA samples; (**b**) leaching test of plasticized PLA samples in *n*-hexane.

It is known that PLA compositions with excellent thermal stability could enhance the end-use properties [35]. Thermal stability testing was conducted on PLA samples blended with various plasticizers. The thermal properties of the PLA samples are presented in Figure 5a and Table S2. Thermal stability test results indicated that the thermal decomposition temperature (*T*_d, 5%_) follows this order: PLA/DOTP < PLA/HDA-2 < PLA/HDA-4 < PLA/HDA-6 < PLA/HDA-8. PLA/DOTP had a significantly lower *T*_d, 5%_ compared to PLA/HDA-2*n*, demonstrating the superior thermal stability of PLA/HDA-2*n* over PLA/DOTP. Firstly, the polar groups within the HDA-2*n* structure form an interaction force with PLA, enhancing their compatibility and, thus, restraining the volatilization of HDA-2*n*. Secondly, the cyclic structure, high molecular weight and the absence of carbon-carbon double bonds in HDA-2*n* further contributed to enhancing the thermal stability of PLA/HDA-2*n*. Furthermore, as the number of ethoxy units in the HDA-2*n* structure increases, the compatibility and thermal stability of PLA/HDA-2*n* also increase, which also increases the thermal stability of PLA blended with HDA-2*n*.

In order to further study the effects of DOTP and HDA-8 on the thermal stability of PLA, the Kissinger method [36] was used to study the non-isothermal thermal degradation kinetics of PLA/DOTP and PLA/HDA-8, and we calculated the thermal degradation activation energy *E* and pre-exponential factor *A*. The calculation results show that the thermal degradation activation energy of PLA/HDA-8 is 79.02 kJ/mol higher than that of PLA/DOTP, indicating that compared with DOTP, the addition of HDA-8 could effectively improve the heat resistance of PLA materials. The calculation results are shown in Figures S3 and S4 and Table S1.

Small molecules of plasticizers in plastics can easily migrate from PLA products to the environment, harming human health, so the leaching test of plasticizers is another important index to evaluate the adaptation of plasticizers to the environment. The migration resistance of plasticizers can determine the scope of application for plasticizers [37]. Figure 5b shows that HDA-2*n* and DOTP-plasticized PLA products in n-hexane solution could be used to simulate the contact process between plasticizer and oily packaging materials [38]. Under the same simulated conditions, the migration of PLA samples (PLA/DOTP) plasticized by traditional petroleum-based plasticizer DOTP was significantly higher than that of PLA samples plasticized by HDA-2*n* (PLA/HDA-2, PLA/HDA-4, PLA/HDA-6 and PLA/HDA-8). Even if soaked in n-hexane solution for 2400 min, only a small amount of HDA-8 was precipitated from the plasticized PLA sample, thanks to the large molecular weight of HDA-8 and the good compatibility of the PLA matrix [30]. To sum up, HDA-8 is more suitable for the application of oil-based commodities than the traditional petroleum-based plasticizer, DOTP.

### 2.6. Volatility Test of Plasticized PLA

**Figure 6:**
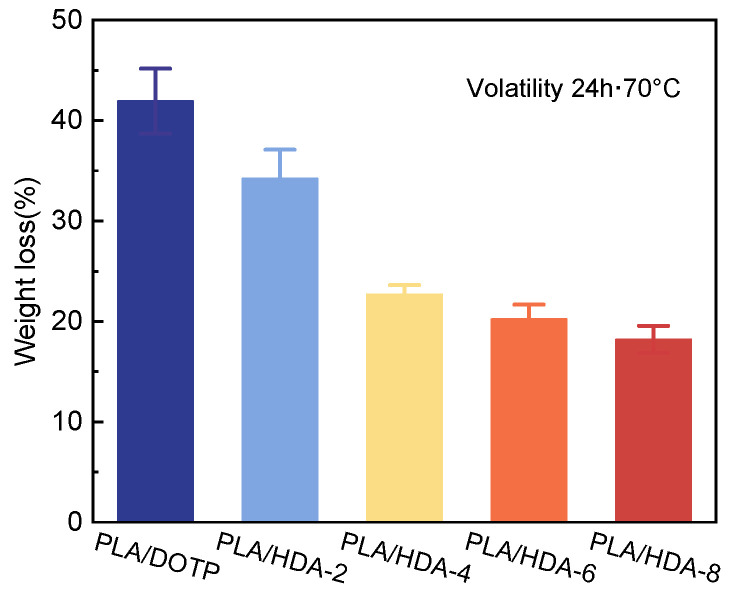
Volatility test of plasticized PLA.

Figure 6 presents the results of volatility tests conducted on PLA products plasticized with hydrogenated dimer acid-based polyethylene glycol methyl ether ester and DOTP. Figure 6 reveals that PLA/HDA-2*n* exhibited superior volatility resistance to that of PLA/DOTP, which could be ascribed to the fact that HDA-2*n* possesses a higher molecular weight than DOTP, a factor that contributes to enhancing the volatility resistance of plasticized products. With an increasing number of ethoxy units, these units promote compatibility between HDA-2*n* and PLA, consequently bolstering the volatility resistance of HDA-2*n*. When compared to PLA/DOTP, PLA/HDA-8 exhibited a 23.74% reduction in volatility, implying that PLA products plasticized with HDA-2*n* were better suited for higher-temperature environments than their counterparts plasticized with traditional DOTP. These findings align with the results of the thermal stability tests.

### 2.7. Water Vapor Transmittance Rate (WVTR) and Oxygen Permeability (OP)

**Figure 7:**
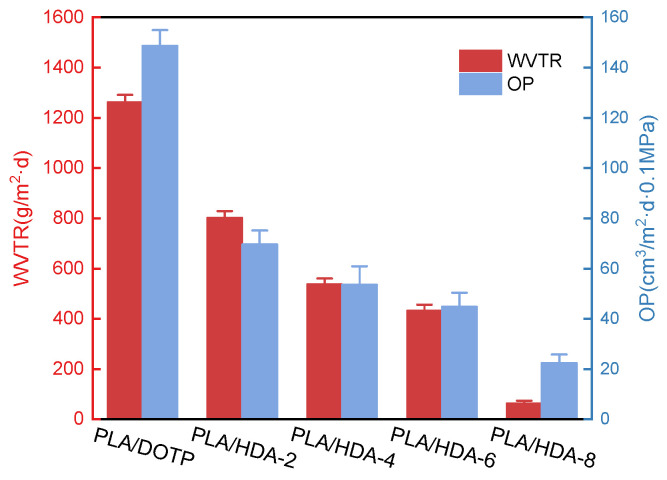
Schematic diagram of the barrier properties of PLA samples to water vapor and oxygen.

The versatility and availability of PLA make it a viable alternative to traditional plastics for packaging uses, such as trays, bottles and cups [39]. Enhancing the barrier properties of paper packaging to water vapor and oxygen is crucial because the presence of water vapor and oxygen in the surrounding air can have an impact on the packaged products [40]. In this work, we conducted an analysis of the water vapor transmittance (WVTR) and oxygen permeability (OP) for the plasticized PLA films. As shown in Figure 7, the WVTR values of plasticized PLA decreased as follows: PLA/DOTP > PLA/HDA-2 > PLA/HDA-4 > PLA/HDA-6 > PLA/HDA-8. PLA/HDA-8 exhibits a WVTR value of 63.24g/m^2^·d, significantly lower than that of PLA/DOTP (1262.1 g/m^2^·d), suggesting that HDA-8 possessed excellent water vapor barrier performance. Similarly, the oxygen permeability displays the same trend, with PLA/DOTP exhibiting an OP value of 148.7 cm^3^/m^2^·d·0.1 MPa, significantly higher than PLA/HDA-8’s value of 22.42 cm^3^/m^2^·d·0.1 MPa. Furthermore, as the quantity of ethoxy units in the HDA-2*n* plasticizer increases, there is a gradual reduction in the WVTR and OP values of the PLA samples, leading to enhanced water vapor and oxygen barrier properties. The underlying mechanism explaining the outstanding water vapor and oxygen barrier properties of PLA/HDA-8 can be summarized as follows: the ethoxy units within HDA-2*n* establish interactions with PLA molecules, fostering a closer connection between the plasticizer and PLA molecular chains. In a word, PLA/HDA-8 exhibits remarkable water vapor and oxygen barrier performance, highlighting its significant advantages for applications within the PLA packaging industry.

### 2.8. Biodegradation Test of Plasticized PLA

**Figure 8:**
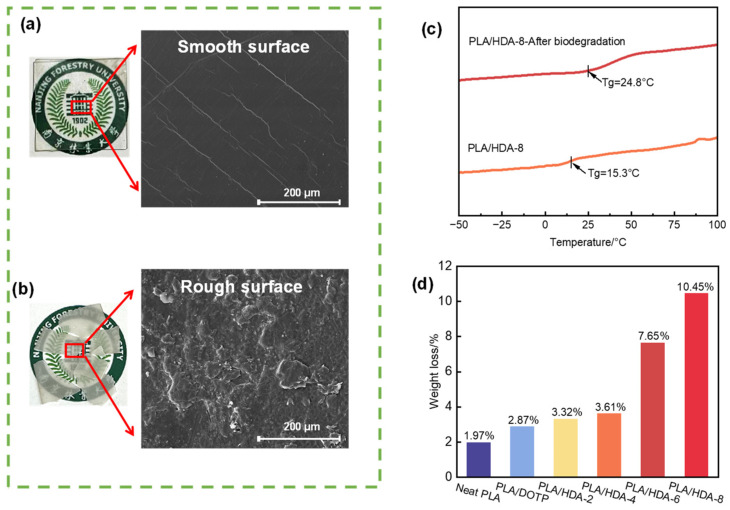
Optical images and SEM of PLA samples before (**a**) and after (**b**) biodegradation behavior in garden soil; (**c**) DSC curve test for the biodegradation process of PLA/HDA-8; (**d**) weight loss of PLA samples after biodegradation behavior.

Biodegradation experiments were conducted on PLA samples blended with various plasticizers. Figure 8a and b display optical images and micro-morphologies of PLA plasticized with HDA-8, both before and after biodegradation. It was observed that PLA samples plasticized with HDA-8 underwent significant changes, both in terms of macroscopic and microscopic aspects. Macroscopically, the transparent film of the HDA-8-plasticized PLA sample exhibited noticeable whitening after undergoing the biodegradation experiment. Microscopically, the surface of the HDA-8-plasticized PLA sample displayed evident erosion after biodegradation, transitioning from a smooth to a markedly rough texture. These findings align well with the DSC test results (Figure 8c), where the glass transition temperature of PLA/HDA-8 increased after the biodegradation experiment. This result could be attributed to the precipitation of the plasticizer in the process of biodegradation. Furthermore, the weight loss rate of PLA samples plasticized with HDA-2*n* after biodegradation (Figure 8d) surpassed that of PLA samples plasticized with the petroleum-based plasticizer, DOTP. Among these, PLA/HDA-8 exhibited the most notable weight loss rate. This conclusion is further supported by the weight loss data obtained from GPC measurements (Figure S5). It can be seen that the molecular weight of pure PLA did not change much, while the molecular weight of PLA/HDA-8 decreased significantly, which further evidenced that HDA-8 promoted the biodegradation of plasticized PLA.

## 3. Materials and Methods

### 3.1. Materials

Hydrogenated dimer acid was obtained from Anhui Hongtai New Material Technology Co., Ltd. (Anqing, China). Ethylene glycol monomethyl ether, diethylene glycol monomethyl ether, triethylene glycol monomethyl ether and tetraethylene glycol monomethyl ether were supplied by Jiangsu Yida Chemical Co., Ltd. (Wuxi, China). Poly(lactic acid) was purchased from Chinese Academy of Science (Institute of Solid State Physics, HFIPS, Hefei, China). Cyclohexane (≥99.0%), p-toluenesulfonic acid (≥98.5%), dioctyl terephthalate (DOTP, ≥99.0%), dichloromethane (99.5%) and sodium carbonate (AR grade) were purchased from Sinopharm Chemical Reagent Co., Ltd. (Shanghai, China). All the materials were used directly, without any purification.

### 3.2. Synthesis of Hydrogenated Dimer Acid-based Polyethylene Glycol Methyl Ether Ester

The structure of hydrogenated dimer acid can be divided into open-chain type, single-ring type and double-ring type. The industrial-grade hydrogenated dimer acid is mainly composed of monocyclic type [41]. In this study, monocyclic hydrodimer acid is selected as the representative.

Hydrogenated dimer acid and alcohol ether (ethylene glycol methyl ether, diethylene glycol methyl ether, triethylene glycol methyl ether and tetraethylene glycol methyl ether) were added to a four-port flask with a water separator and condensation tube at a molar ratio of 1:2.5. Then, 1% *p*-toluenesulfonic acid (based on the mass of hydrogenated dimer acid, the same as below) and 40% cyclohexane were gradually heated to 120 °C and reacted for 7 h. The reaction ends when the acid value is less than or equal to 1 mgKOH/g. The unreacted cyclohexane and ethylene glycol ether were removed by refining, and the remaining liquid was adjusted to neutral with 10% Na_2_CO_3_, then washed with distilled water, and the hydrogenated dimer acid-based polyethylene glycol methyl ether ester (HDA-2*n*) was prepared by vacuum distillation.

### 3.3. Preparation of PLA Samples

**Scheme 1:**
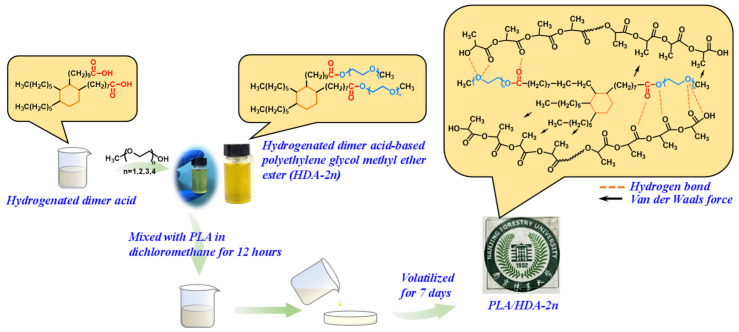
Synthesis of hydrogenated dimer acid-based polyethylene glycol methyl ether ester and preparation of PLA samples.

As shown in Scheme 1, the solvent casting method was employed to prepare PLA samples. In the initial step, PLA was mixed with a 20 wt% concentration of plasticizers, including HDA-2, HDA-4, HDA-6 and HDA-8. The resulting mixture underwent thorough dissolution in a dichloromethane solvent to attain 10% (*w*/*w*) solutions. Subsequently, the prepared solution was poured into a glass Petri dish and placed in an oven set at a constant temperature of 30 °C. This prolonged thermal treatment spanned a duration of 168 h until the sample weight did not change, enabling the complete removal of residual dichloromethane. The resulting PLA samples exhibited a remarkably smooth surface morphology, possessing a uniform thickness of approximately 1.0 mm. Each individual sample was labeled as PLA/HDA-2, PLA/HDA-4, PLA/HDA-6 and PLA/HDA-8, respectively. Moreover, following an analogous procedure, PLA/DOTP was also prepared, adhering to the established methodology.

### 3.4. Characterization

Infrared characterization of liquid and solid samples was conducted using an infrared spectrometer (Nicolet Nexus 670, Thermo Scientific, Waltham, MA, USA) at a 4 cm^−1^ resolution, scanning 64 times per sample, and covering a wavenumber range of 400~4000 cm^−1^. Nuclear magnetic resonance spectroscopy for hydrogenated dimer acid and its ester was performed on a Bruker spectrometer (600 MHz, Billerica, MA, USA), utilizing deuterated chloroform (CDCl_3_) as the solvent.

Tensile property tests were carried out on a universal testing machine (MTSE44.304, MTS, Eden Prairie, MN, USA) according to the GB/T1040.3-2006 standard [42] using dumbbell-shaped splines prepared from PLA sheets. The tensile rate was set to 10 mm/min, testing five specimens per sample.

Glass transition temperature measurements were taken using a differential scanning calorimeter (DSC 25, TA Instruments Inc., New Castle, DE, USA) with a 5~10 mg sample under nitrogen atmosphere, temperature range between −80 °C and 120 °C, and heating rate of 5 °C/min.

X-ray diffraction (XRD) of thin-film samples is measured using X-ray diffractometer (Ulima IV, Rigaku, Saitama Prefecture, Japan). The ray source is Cu target Kα ray, the target voltage is 40 kV, the tube current is 30 mA, the scanning rate is 10(°)/min and the scanning range is 2θ = 5~40°.

Scanning electron microscopy (SEM) was employed to examine the spline fracture surfaces (JSM-7600, JEOL, Tokyo, Japan) operating at an accelerating voltage of 15 kV with sample surfaces coated in gold to enhance conductivity. The plasticized PLA samples were tested by a UV-vis spectrophotometer (Model Lambda 25, Perkin-Elmer, Waltham, MA, USA).

Thermal stability tests were performed on a thermogravimetric analyzer (TGA/DSC/1100SF, Mettler-Toledo, Zurich, Switzerland) in a nitrogen atmosphere using 5~15 mg samples at a temperature range of 50~700 °C and a heating rate of 10 °C/min.

Migration resistance tests adhered to the GB/T3830-2008 standard [34], submerging the sample (20 × 20 × 1 mm) in an n-hexane solution at 23 ± 2 °C and 50 ± 10% humidity. Samples were dried at 50 °C for 6 h, and the mass loss rate was determined via Equation (1):

(1) $$\mathrm{Migration}\;\mathrm{degree}=\frac{W_{\mathrm{before}}-W_{\mathrm{after}}}{W_{\mathrm{before}}\;\times \;x}\times 100\%$$

where *W_before_* and *W_after_* represent the weight of the PLA samples before and after the test, respectively, and *x* represents the initial concentration of plasticizer in the PLA sample, testing five samples per set.

Volatility resistance tests were conducted on plasticized PLA samples (20 × 20 × 1 mm) by placing them in an oven at 70 °C for 24 h. Following this, samples were cooled to room temperature, and the mass loss rate was calculated using Equation (1) based on the mass change before and after the test, with five samples tested per batch.

Water vapor transmission rate (WVTR) analysis was performed on a 74 mm diameter PLA sample at a temperature of 38 ± 0.6 °C and relative humidity of 50 ± 2% using a W3-031 water vapor transmittance tester (W3/036, Labthink, Jinan, China). The PLA samples’ oxygen permeability (OP) was determined in accordance with GB/T 19789-2021 [43] using a differential pressure gas permeameter (VAC-V1, Labthink, Jinan, China). Testing was conducted at 23 ± 2 °C with a relative humidity of 50 ± 10%. A minimum of three specimens from each sample were tested to determine the mean value. At least three specimens of each sample were tested to calculate the mean value.

The biodegradation of plasticized PLA was assessed through garden soil burial tests. Initially, samples measuring 20 × 20 × 1 mm were buried in soil at an approximate depth of 8 cm below the soil surface. Subsequently, these specimens were transferred to a temperature and humidity chamber (GDJS-50A, Shanghai Hecheng Instrument Co., Shanghai, China) set to maintain 40% relative humidity at 25 °C for a duration of 90 days. Finally, the specimens underwent vacuum drying at 25 °C. The weight losses were obtained before and after the biodegradation, according to Equation (2).

(2) $$\mathrm{Biodegradation}\;\mathrm{degree}=\frac{W_{\mathrm{before}}-W_{\mathrm{after}}}{W_{\mathrm{before}}}\times 100\%$$

where *W_before_* and *W_after_* represent the weight of the PLA samples before and after the test. The relative molecular weight and distribution of biodegradable samples before and after biodegradation were determined by Agilent PLGPC220 gel permeation chromatography (GPC) with tetrahydrofuran as mobile phase at 35 °C. The surface morphology of the samples before and after biodegradation was observed by scanning electron microscope system (SEM), determining the glass transition temperature of samples after biodegradation using a differential scanning calorimeter (DSC).

## 4. Conclusions

By utilizing non-toxic hydrogenated dimer acid and polyethylene glycol methyl ether as raw materials, we successfully synthesized four variants of hydrogenated dimer acid-based polyethylene glycol methyl ether esters (HDA-2*n*, 2*n* = 2, 4, 6 or 8 referring to the ethoxy units) through a one-step method. These esters were subsequently employed for plasticizing PLA, and the effects of HDA-2*n* on the PLA properties were systematically investigated. The results indicate a direct correlation between the plasticizing efficacy of HDA-2*n* and the number of introduced flexible ethoxy units. As the quantity of ethoxy units increased, the compatibility between HDA-2*n* and PLA improved, leading to significant enhancements in the mechanical properties, thermal stability, migration resistance, as well as volatility resistance of PLA. In comparison to PLA plasticized with DOTP, PLA/HDA-8 demonstrated superior low-temperature resistance (40.1 vs. 15.3 °C), enhanced tensile properties (elongation at break: 316.96 vs. 598.28%), elevated thermal stability (246.8 vs. 327.6 °C), improved gas barrier properties, and enhanced biodegradability. The results unequivocally demonstrated that the overall performances of HDA-8, processing the highest number of ethoxy groups, were better than for other plasticizers. These findings underscore the potential of HDA-8 as a comprehensive alternative to the conventional petroleum-based plasticizer, DOTP.

## Figures and Tables

**Table 1:** Comparison between this work and other research on plasticizing PLA.

Plasticizer	Elongation at Break (%)	*T*_g_ (°C)	Reference
Epoxidized palm oil (EPO)	88	62.7	Chieng et al. [27]
Dipropyl maleate (DPM)	200	5.0	Enumo et al. [28]
Isosorbide dioctoate (SDO)	281	32.5	Yang et al. [29]
Biosourced glycerol plasticizer (GS-C4-20)	435	24.0	Halloran et al. [30]
Acetylated malic acid alkyl esters (AcMAE-C6)	567	28.8	Park et al. [31]
This work (HDA-8)	598	15.3	-

## Data Availability

All data are included in the manuscript and Supplementary Materials.

## Supplementary Materials

The following supporting information can be downloaded at: https://www.mdpi.com/article/10.3390/molecules29112526/s1, NMR data of HDA and HDA-2*n*; Figure S1: ^13^C NMR spectra of HDA and HDA-2*n*; Figure S2: Possible interaction between HDA-2*n* and PLA matrix; Figure S3: TG and DTG curves of PLA/DOTP and PLA/HDA-8 at different heating rates; Figure S4: ln(*β*/*T*_P_^2^)-*T*_P_^−1^ curves; Figure S5: GPC test for the biodegradation process of neat PLA and PLA/HDA-8; Table S1: TGA kinetic data of PLA/DOTP and PLA/HDA-8 at different heating rates; Table S2: Thermal stability of plasticized PLA determined by TG.
