# Supplementary material for: Biodegradable Hydrogenated Dimer Acid-Based Plasticizers for PLA with Excellent Plasticization, Thermal Stability and Gas Resistance
Source: Molecules. 2024 May 27;29(11):2526. doi: 10.3390/molecules29112526 (PMC11173700; doi:10.3390/molecules29112526)
Supplement: Supplementary file 1 [file molecules-29-02526-s001.zip › molecules-3005828-supplementary.pdf]

Supporting Information

CONTENT

S1. NMR characterization of hydrogenated dimer acid (HDA) and hydrogenated dimer acid-based polyethylene glycol methyl ether ester (HDA-2*n*)········3
S2. ^{13}C NMR spectra of HDA and HDA-2*n*········3
S3. Possible interaction between HDA-2*n* and PLA matrix········4
S4. TGA kinetic reaction kinetics········4
S5. GPC test for the biodegradation process of neat PLA and PLA/HDA-8········5
S6. Thermal stability of plasticized PLA determined by TG ········6

S1. NMR characterization of hydrogenated dimer acid (HDA) and hydrogenated dimer acid-based polyethylene glycol methyl ether ester (HDA-2n)

HDA: ^{1}H NMR (600 MHz, Chloroform-*d*) δ 2.35 (t, 4H), 1.63 (p, 4H), 1.41 – 1.11 (m, 39H), 0.90 – 0.82 (m, 6H). ^{13}C NMR (151 MHz, Chloroform-*d*) δ 180.51 , 34.13 , 31.95 , 30.16 , 29.73 – 28.66 (m), 24.68 , 22.71 , 14.13 .

HDA-2: ^{1}H NMR (600 MHz, Chloroform-*d*; *δ*, ppm) δ 4.28 – 4.17 (m, 4H), 3.57 (m, 4H), 3.37 (d, 6H), 2.32 (t, 4H), 1.61 (t, 6H), 1.24 (q, 50H), 0.86 (t, 6H); ^{13}C NMR (151 MHz, Chloroform-*d*) δ 173.57 , 70.26 , 62.97 , 58.69 , 33.91 , 31.66 , 29.41, 29.21 , 29.00 , 28.86 , 24.65 , 22.42 , 13.85 .

HDA-4: ^{1}H NMR (600 MHz, Chloroform-d; δ, ppm) δ 4.21 (dd, 4H), 3.73 – 3.65 (m, 4H), 3.64 – 3.51 (m, 8H), 3.38 – 3.35 (m, 6H), 2.31 (t, 4H), 1.60 (p, 6H), 1.45 – 0.90 (m, 45H), 0.88 – 0.81 (m, 6H);13C NMR (151 MHz, Chloroform-d) δ 173.82 , 71.88 , 70.46 , 69.24 , 63.29 , 59.04 , 34.20 , 31.92 , 29.67 , 29.47 , 29.27 , 29.13 , 24.90 , 22.68 , 14.11 .

HDA-6: ^{1}H NMR (600 MHz, Chloroform-*d*; *δ*, ppm) δ 4.20 (dd, 4H), 3.67 (dd, 4H), 3.64 – 3.50 (m, 16H), 3.36 (s, 6H), 2.30 (t, 4H), 1.59 (q, 6H), 1.26 (s, 47H), 0.88 – 0.82 (m, 6H). ^{13}C NMR (151 MHz, Chloroform-*d*) δ 173.80 , 71.92 , 69.20 , 63.33 , 59.01 , 34.20 , 31.91 , 29.58 , 29.21 , 24.90 , 14.11 .

HDA-8: ^{1}H NMR (600 MHz, Chloroform-d; δ, ppm) δ 4.23 – 4.19 (m, 4H), 3.67 – 3.60 (m, 24H), 3.35 (s, 6H), 2.30 (d, 4H), 1.59 (t, 6H), 1.18 (s, 48H), 0.85 (tt, 6H). 13C NMR (151 MHz, Chloroform-d) δ 71.92 , 70.58 , 69.19 , 63.34 , 34.20 , 31.91 , 29.67 , 29.28 , 29.14 , 24.90 , 22.68 , 14.11 .

S2. ^{13}C NMR spectra of HDA and HDA-2n

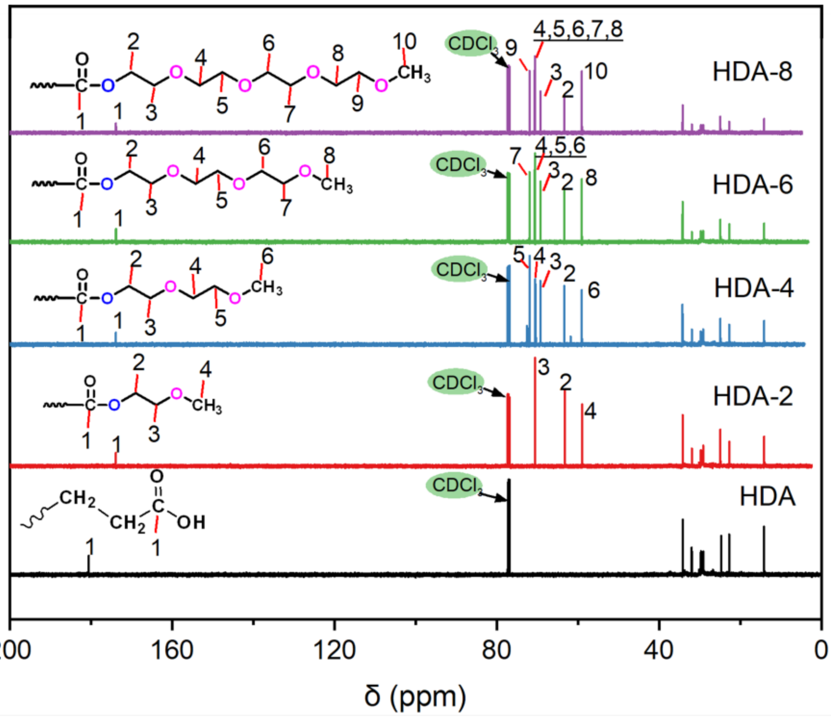

Figure S1. ^{13}C NMR spectra of HDA and HDA-2*n*

S3. Possible interaction between HDA-2n and PLA matrix

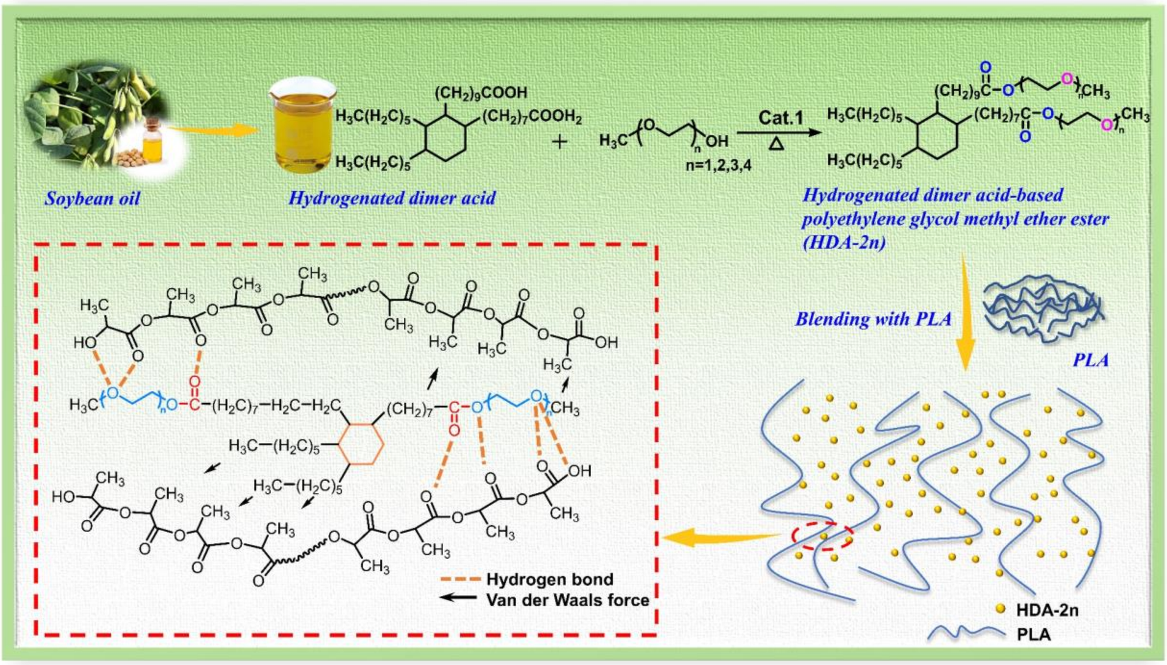

Figure S2. Possible interaction between HDA-2*n* and PLA matrix

S4. TGA kinetic reaction kinetics

Expression of Kissinger method:

$$\ln\left(\frac{\beta}{T_P^2}\right) = \ln\left(\frac{AR}{E}\right) - \frac{E}{RT}$$

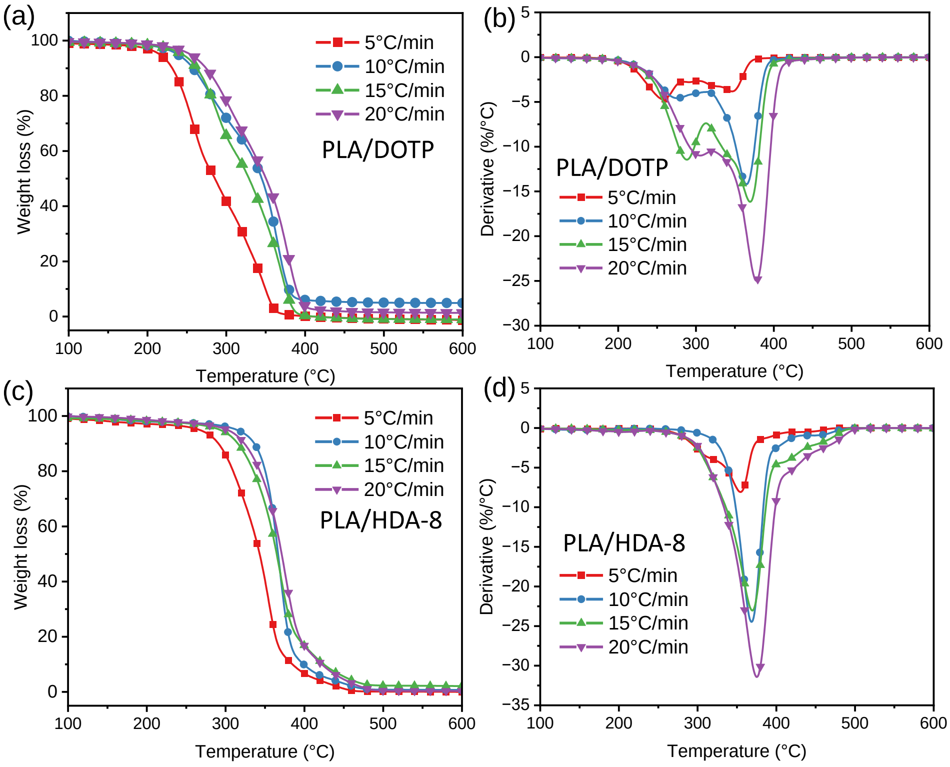

Figure S3. TG and DTG curves of PLA/DOTP and PLA/HDA-8 at different heating rates

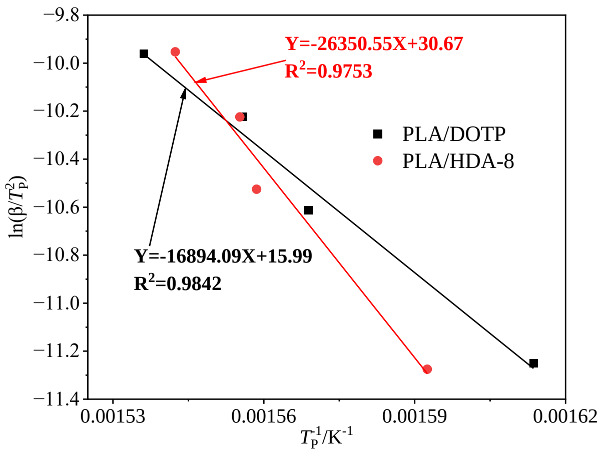

Figure S4. $\ln(\beta/T_P{}^2)$-$T_P{}^{-1}$ curves

Table S1. TGA kinetic data of PLA/DOTP and PLA/HDA-8 at different heating rates

Samples	β/°C·min^{-1}	T_P/K	$T_P{}^{-1}$/K^{-1}	$\ln(\beta/T_P{}^2)$	E/kJ·mol^{-1}	A/min^{-1}
PLA/DOTP	5	619.73	1.6136×10^{-3}	-11.2491	140.06	1.48×10^{8}
	10	637.40	1.5689×10^{-3}	-10.6122		
	15	642.76	1.5558×10^{-3}	-10.2234		
	20	650.97	1.5362×10^{-3}	-9.96119		
PLA/HDA-8	5	627.94	1.5925×10^{-3}	-11.2754	219.08	5.50×10^{14}
	10	641.61	1.5586×10^{-3}	-10.6253		
	15	642.99	1.5552×10^{-3}	-10.2242		
	20	648.34	1.5424×10^{-3}	-9.95310		

S5. GPC test for the biodegradation process of neat PLA and PLA/HDA-8

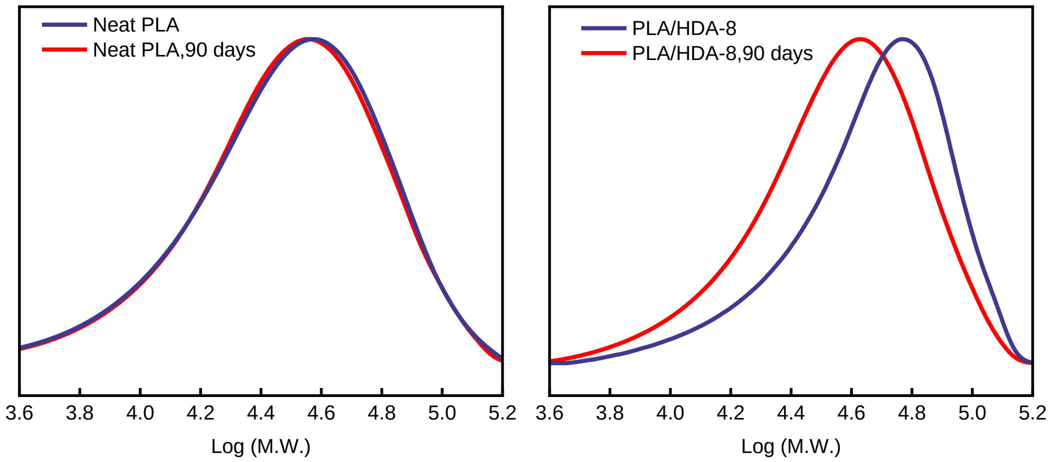

Figure S5. GPC test for the biodegradation process of neat PLA and PLA/HDA-8

S6. Thermal stability of plasticized PLA determined by TG

Table S2. Thermal stability of plasticized PLA determined by TG

**Samples**	$T_{d,5\%}$ (°C)	$T_{d,10\%}$ (°C)	$T_{d,50\%}$ (°C)
PLA/DOTP	246.8	274.7	356.2
PLA/HDA-2	301.5	317.5	353.7
PLA/HDA-4	308.7	323.7	358.5
PLA/HDA-6	319.1	333.9	361.4
PLA/HDA-8	327.6	341.2	365.8
